# Auditory Cues Alter the Magnitude and Valence of Subjective Sexual Arousal and Desire Induced by an Erotic Video

**DOI:** 10.1007/s10508-023-02802-4

**Published:** 2024-02-01

**Authors:** James G. Pfaus, Ellen Zakreski

**Affiliations:** 1 Department of Psychology and Life Sciences, Faculty of Humanities, Charles University, 18200 Prague, Czech Republic; 2 Center for Sexual Health and Intervention, Czech National Institute of Mental Health, Klecany, Czech Republic

**Keywords:** Subjective sexual arousal and desire, Visual, Auditory, Erotic video, Ejaculation

## Abstract

Although women and men rate their subjective arousal similarly in response to “female-centric” erotic videos, women rate their subjective arousal lower than men in response to “male-centric” videos, which often end with the male’s ejaculation. This study asked whether ratings of subjective sexual arousal and desire using the Sexual Arousal and Desire Inventory (SADI) would be altered if this ending was present or absent, and whether including or excluding the accompanying soundtrack would influence the magnitude and direction of the responses. A total of 119 cis-gendered heterosexual undergraduates (59 women and 60 men) viewed an 11-min sexually explicit heterosexual video that ended with a 15-s ejaculation scene. Two versions of the video were created, one with the ejaculatory ending (E+) and one without (E−). Participants were assigned randomly to view one of the two versions with (S+) or without (S−) the accompanying soundtrack, after which they completed the state version of the SADI. Women and men found both sequences without sound less arousing on the Evaluative, Motivational, and Physiological subscales of the SADI relative to the S+ sequences. However, on the Negative/Aversive subscale, women found the E + S- sequence more negative than did men, whereas this difference was not found with sound. Thus, women and men were sensitive to the auditory content of sexually explicit videos, and scenes of sexual intercourse ending with explicit ejaculation increased the Evaluative and Motivational properties of subjective sexual arousal and desire. However, this occurred in women only when the auditory cues signaled a clear and gratifying sexual interaction.

## Introduction

Sexual arousal and desire involve both automatic and higher order cognitive responses to erotic cues (Janssen et al., 2000). Sexual or general autonomic arousal can be assessed physiologically from measures of genital blood flow or galvanic skin response in women and men (Hoon et al., 1976; Janssen, 2011; Laan & Everaerd, 1998; Rowland, 2005; Wenger et al., 1968), and also from subjective ratings (Conte, 1983; Meston & Derogatis, 2002; Rosen, 1998; Toledano & Pfaus, 2006). Both sexual arousal and desire can be provoked as states in response to the presentation of erotic images, videos, auditory recordings, narratives, and fantasy or can be regarded as traits that can be measured with questions about one’s own subjective perception of normal functioning. For both women and men, habituation to visual sexual stimuli (VSS) occurs when stimuli of sufficient arousing potential are presented repeatedly (Both et al., 2011; Dawson et al., 2012; Koukounas & Over, 2001; Meuwissen & Over, 1990), and dishabituation occurs when the stimuli or context of the stimuli are altered unexpectedly after repeated presentations (Both et al., 2011; Meuwissen & Over, 1990; Rupp & Wallen, 2008).

Despite many similarities between women and men in physiological sexual arousal and brain responses to VSS, there are some notable differences. For example, using photoplethysmography to assess genital blood flow, men typically display concordance in their genital and subjective responses to VSS whereas women do not (Chivers & Bailey, 2005; Chivers et al., 2004, 2010; Suschinsky et al., 2009). However, genital and subjective responses become more concordant in women with more sexual experience and familiarity with their own arousal and desire, which together may vary with sexual orientation (Chivers et al., 2015; Meston & Stanton, 2019). Men’s genital responses tend to be more category-specific compared to women, whereas women’s genital responses are described as more “fluid,” being provoked by a variety of VSS scenarios and contexts (Huberman et al., 2015). Similar data can be found in eye-tracking studies, where first saccades and gaze time toward particular aspects of VSS is concordant with genital responses in men, but not in women (Lykins et al., 2008; O'Kane et al., 2023), although attention toward particular VSS in women can be diminished with oral contraceptive use (Rupp & Wallen, 2007) and altered depending on the phase of the menstrual cycle during the first exposure (Wallen & Rupp, 2010). In contrast, although explicit VSS provoke greater genital arousal in both women and men compared to romantic visual stimuli, women instructed to fantasize about their partners had greater subjective arousal during the presentation of explicit VSS compared to women instructed to fantasize about someone else (Carvalho et al., 2013). This suggests that sexual arousal in women also depends on conscious awareness of stimulus characteristics and relationship context.

Differences also exist between women and men in the subjective sexual arousal induced by videos that are either made for a female or male audience, and thus deemed “female-centric” or “male-centric,” respectively (Laan et al., 1994). Heterosexual female-centric videos often spend more time depicting kissing, erogenous touching, clitoral stimulation, and female orgasms, relative to heterosexual male-centric videos, which spend more time depicting penile stimulation, vaginal intercourse, and especially male ejaculation. Janssen et al. (2003) assessed the subjective sexual arousal induced by 20 video clips (each 3 min in duration), half of which were female-centric and the other half male-centric. Although subjective sexual arousal was higher overall for men compared to women, women rated their sexual arousal to the female-centric clips higher than the male-centric clips, whereas men rated their sexual arousal to the male-centric clips higher than the female-centric clips.

Understanding how different components of erotic stimuli affect arousal and desire in men and women is important as it allows researchers to better select stimuli to induce sexual responses in their participants. Most sexually explicit videos used as experimental stimuli contain auditory soundtracks that typically amplify the impact of visual cues and interactions through music, dialog, and/or the emotional utterances of the actors. Presumably, this helps people become absorbed emotionally into the video (Kwon et al., 2022). However, despite this, few studies have examined the role of auditory stimuli during sexual arousal or desire. Auditory sexual cues (ASCs), such as moaning, panting, or verbal expressions of pleasure, have been used to provoke genital and subjective sexual arousal in men (Hall et al., 1985; O’Donohue & Plaud, 1991) and women (Chivers & Timmers, 2012), although in women, the presence or absence of ASCs during early genital sexual arousal with VSS (e.g., within the first 2 min) did not alter the magnitude of vaginal blood flow (Polan et al., 2003). As with VSS, men’s genital responses to ASCs showed category specificity whereas women’s genital responses did not (Chivers & Timmers, 2012). These data support a difference between women and men in their responsiveness to both visual and auditory sexual cues, and it may be the case that early phases of genital sexual arousal depend more on automatic processing (Janssen et al., 2000) which, in women, may reach a maximum with VSS alone. It has been suggested that ASCs, relative to VSS, are processed more slowly and at a higher order cognitive level (Gaither & Plaud, 1997), and that ASCs can alter the overall perception of VSS by altering the perceived emotional intensity or quality of the action depicted in the video. It was proposed that ASCs facilitate sexual arousal through a two-step cognitive process that involves stimulus appraisal and subsequent translation into visual imagery or summation with the type of VSS present in a video (Przybyla & Byrne, 1984; Przybyla et al., 1983). In one study, attractive versus unattractive faces were paired with an erotic audio sequence and viewed by men who rated their subjective level of sexual arousal (Hawk et al., 2007). The erotic audio sequence facilitated the rate at which men reported peak subjective sexual arousal to pictures of attractive faces relative to no face, but when paired with unattractive faces, the audio sequence extended the latency to report sexual arousal and reduced the magnitude of peak arousal. ASCs may thus moderate how VSS are processed and affect the conscious awareness of arousal and desire. However, altering an erotic video’s soundtrack from active rock music and dialog (including ASCs) to relaxing background music alone diminished the subjective negative ratings of “pornographic content” significantly but did not alter subjective sexual arousal in men (Pfaus et al., 1986).

Although ACSs and other auditory stimuli in erotic videos appear to contribute to the overall sexual and emotional impact, it is not clear if this impact is similar for women and men, or whether the male-centric versus female-centric nature of the sexual action is altered differently by ACSs. In the study by Janssen et al. (2003), one of the video clips was rated as inducing relatively equal, but moderate, levels of sexual arousal in both women and men (film #3, p. 246). This clip was unique in that it was taken from a largely female-centric video that ended with a male-centric scene of explicit male ejaculation. This video provides an opportunity to ask several questions about the erotic value of ASCs and depicted male ejaculation: What contribution does the soundtrack make to subjective assessments of sexual arousal and desire provoked by the video? What contribution does the explicit ejaculation scene make to those assessments? Do they differ between women and men? And do they interact? To accomplish this, we altered the full-length video so that it was presented with or without sound, and with or without the explicit ejaculation scene, to different groups of women and men. Subjective assessments were made using the Sexual Arousal and Desire Inventory (SADI; Toledano & Pfaus, 2006). The SADI is a validated, adjective-based scale that includes Evaluative, Motivational, Physiological, and Negative/Aversive subscales that reflect multidimensional aspects of the overall subjective experience (Bosch et al., 2017; Dolder et al., 2018; Persson et al., 2016; Toledano & Pfaus, 2006). If the soundtrack adds to the emotional and sexual absorption, then given the data of Hawk (2007), Przybyla and Byrne (1984), and Przybyla et al. (1983), we hypothesized that the video without sound would be rated lower on the Evaluative, Motivational, and Physiological subscales and higher on the Negative/Aversive subscale by women and men. Likewise, if the ejaculation scene at the end is more male-centric, then given the data of Janssen et al. (2003), the women should rate the video with the ejaculation scene lower on the Evaluative, Motivational, and Physiological subscales and perhaps higher on the Negative/Aversive subscale, relative to men.

## Method

### Participants

Self-identified heterosexual undergraduate women (*N* = 80) and men (*N* = 80) at a metropolitan university in Montréal, QC, Canada, were recruited as part of their required participation in an undergraduate research subjects pool. Participants ranged in age from 19 to 28 years. The study was listed under the title, “Stimuli that provoke sexual arousal and desire,” on a website that contained titles, brief descriptions, and contact information of studies open to participation in the subject pool. Participants contacted researchers by email and were sent a form that described the study and included questions about age, sex, gender, sexual orientation, sexual experience, and current contraceptive and medication use. An informed consent form was also included that stated how participant data would be pooled into means and standard errors and that their participation in this particular study would be kept completely confidential. Participants that sent back the questionnaire with a signed informed consent form were then scheduled for a 30-min test at their convenience. Only participants that self-identified as cis-gendered, heterosexual, or mostly heterosexual (i.e., scored < 3 on the Kinsey scale; Kinsey et al., 1948, 1953), that were sexually active, that had prior experience viewing sexually explicit material, and that were not currently on prescription medication except oral contraceptives, were enrolled. Participants received full subject pool credit after completing the test. Of the participants enrolled, 59 women and 60 men returned complete data.

### Measures

#### Stimuli

An 11-min video entitled “Under a Gazebo” was adapted with permission from the collection *Outdoor Ecstasy* (2001; Scene One, Adam & Eve Productions, Beverly Hills, CA; available at https://www.adultdvdempire.com/1650750/outdoor-ecstacy-porn-videos.html). The video was chosen on the basis of the study by Janssen et al. (2003) that assessed the arousing potential of different sexually explicit videos in women and men. The video depicted a presumably Caucasian man and woman outdoors engaging in consensual petting (kissing, genital, and non-genital touching), oral sex, and penile–vaginal intercourse. The action was solicited and directed largely by the female actor. The soundtrack contained her non-verbal emotional vocalizations over a New-Age Celtic instrumental music background. The male actor grunted once at the end of the video during his ejaculation. As mentioned above, the sequence was previously rated in the Janssen et al. study as being equally and moderately arousing for both women and men. The video ended with a scene of explicit extra-vaginal ejaculation on the breasts of the female actor in the last 15 s. This last scene began with a break in the action where the male actor pulled out and moved over to the upper torso of the female actor. We created two versions of the video, one original with the ejaculatory ending (E+) and one that faded to black and ended immediately after the male actor pulled out prior to his ejaculation (E−). Two versions of each of those were created with the sound on (S+) or off (S−) throughout the video. This rendered four videos, the original E+S+, and three variants, E−S+, E+S−, and E−S−, which were used for the study.

#### Subjective Arousal and Desire Ratings

Participants completed the SADI (Toledano & Pfaus, 2006), a 54-item adjective-based self-report measure of sexual arousal and desire that has been validated for use in both females and males. To examine state levels of arousal and desire, participants were asked to rate how well each adjective describes their reaction to an independent treatment in the current moment (e.g., as provoked by pictures or a video). Each adjective was rated on a 6-point Likert scale ranging from 0 (“does not describe me at all”) to 5 (“describes me perfectly”). Option 3 was labeled “describes me moderately well.” The SADI consists of four orthogonal subscales: Evaluative, Physiological, Motivational, and Negative/Aversive, which reflect distinct components of sexual arousal and desire. Different scales have different numbers of items. Scores for each subscale were calculated by summing their respective items.

The Evaluative subscale of the SADI reflects the cognitive/emotional assessment of sexual arousal and desire. It includes 27 descriptors for a total possible score of 135. Example items include “Forget about all else,” “Good,” or “Happy.”

The Physiological subscale reflects the subjective perception of physiological sexual arousal and desire. It is loaded onto by 17 descriptors for a total possible score of 85. Example items on Physiological subscale are “Flushed,” “Heart beats faster,” or “Tingling in genital area.”

The Motivational subscale reflects the subjective perception of wanting sexual stimulation or interaction. It is loaded onto by 10 descriptors for a total possible score of 50. The Motivational subscale includes items such as “Driven,” “Urge to satisfy,” or “Impatient.”

The Negative/Aversive subscale reflects the subjective perception of inhibition, aversion, and disgust. It is loaded onto by 17 descriptors for a total possible score of 85. It includes items such as “Anxious,” “Sluggish,” and “Repulsion.”

### Procedure

Participants were scheduled randomly to view one of the four video sequences in a room with dimmed lighting that contained a comfortable armchair and table with a computer across from a video screen. Before entering the room, participants were instructed to press “Enter” on the computer when they were ready to view the video, and after viewing, to fill out a paper copy of the SADI that was on the table. Participants were asked how well each adjective described how they felt while viewing the video to get an assessment of the state of sexual arousal and desire induced by the video. Upon completion of the SADI, participants were given a debriefing that explained the purpose of the study, and their participation was confirmed in the confidential subject pool log. Thirty-one participants did not fill out the SADI completely and were dropped from the analysis. The final subject groups were E+S+(*N* = 20 females, 20 males), E−S+(*N* = 20 females, 20 males), E+S− (*N* = 9 females, 10 males), and E−S− (*N* = 10 females, 10 males).

### Statistical Analyses

Data from the SADI for each participant were summed into the four subscales and the means evaluated using a 2 (Auditory Condition, S+ vs. S−) × 2 (VSS Condition, E+ vs. E−) × 2 (Gender, female vs. male) between-subjects analysis of variance (ANOVA) that analyzed the three main effects and their two- and three-way interactions for each subscale. Because dropping the incomplete questionnaires from analysis resulted in unequal Ns, we used the O’Brien (1981) test to determine homogeneity of variance for each SADI subscale. None of the tests was significant, indicating that the variance of the two groups was equivalent. For each significant main effect and interaction, post hoc Tukey HSD tests were conducted to compare the simple means, but corrected for unequal Ns using the Spjotvoll/Stoline method, *p* < 0.05. Estimates of effect size were made using eta squared (*η*^2^). Although significance was set two-tailed at *p* < 0.05, it is justified to report effects with *p* values between 0.05 and 0.10 if the *η*^2^ indicates a medium (0.06–0.13) or large (0.14+) effect size (Kline, 2004; Lakens, 2013). G*Power (Faul et al., 2007) was used to determine sample size requirements for a three-way independent groups’ ANOVA. Statistical power was set to 0.80 and alpha set to 0.05. A medium effect is indicated by a *η*^2^ value between 0.06 and 0.13. This requires a sample size of 55–126.

## Results

**Fig. 1:**
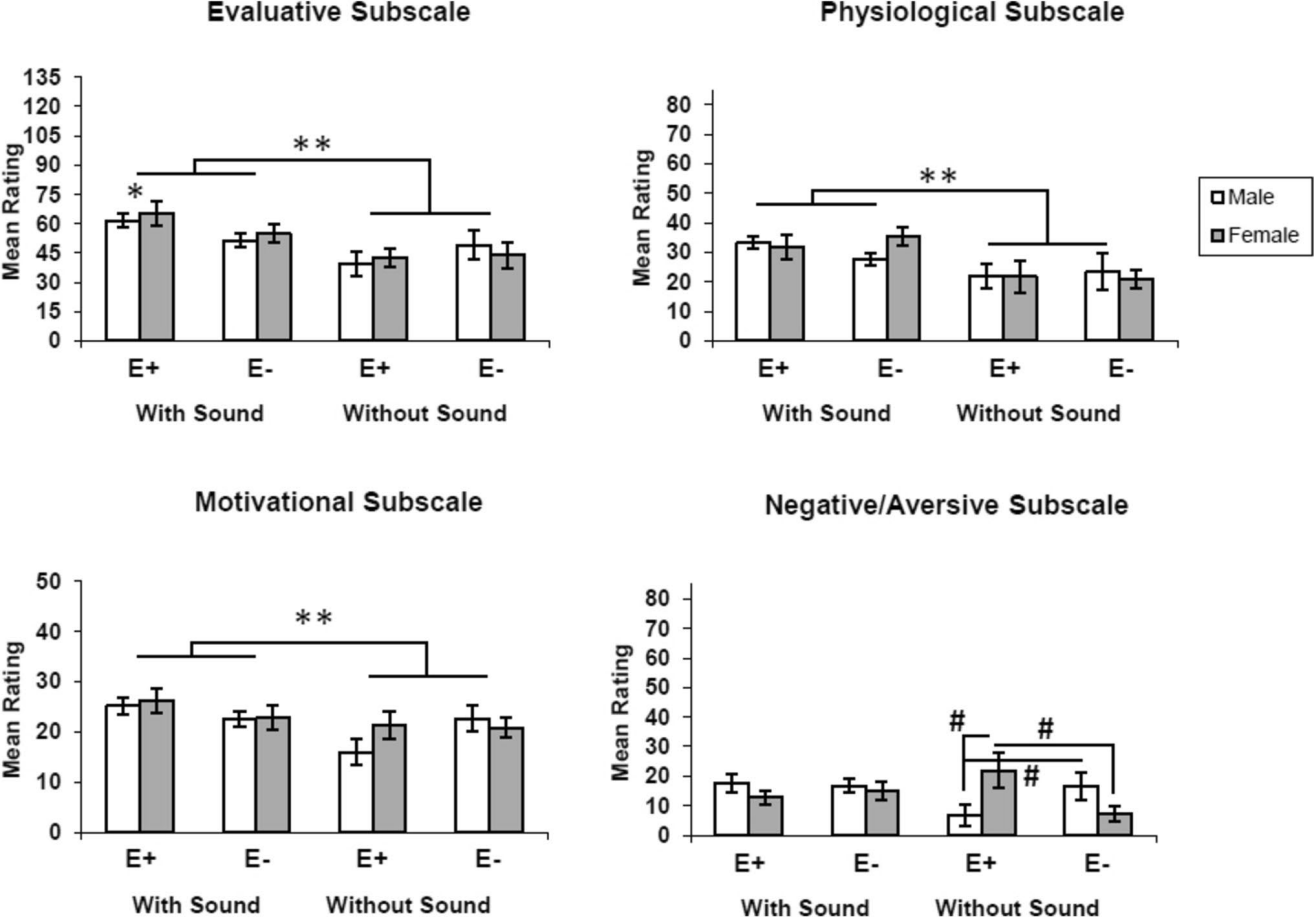
Ratings of the four videos on the Evaluative, Physiological, Motivational, and Negative/Aversive subscales of the Sexual Arousal and Desire Inventory. E+S+: original video with the ejaculatory ending and sound; E−S+ : video with sound but with the ejaculatory ending removed; E+S−: video with the ejaculatory ending and no sound; and E−S−: video without the ejaculatory ending and no sound. *p < 0.05 from the other video sequences across gender, Tukey post hoc test. **p < 0.05, S+ vs. S−, Tukey post hoc test. #*p* < 0.05, E+ vs. E− by gender, Tukey post hoc test

The effects of the four versions of the video on the subscales of the SADI are shown in Fig. 1. In general, participants rated the Evaluative, Physiological, and Motivational subscales in the middle of the total possible range, similar to the overall rating of moderate subjective sexual arousal found for this video by Janssen et al. (2003). The Negative/Aversive ratings were at the lower end of the total possible range, indicating that the video was perceived overall as positive.

### Evaluative Subscale

Table 1 shows the results of the ANOVA for the Evaluative subscale ratings of the four videos by women and men. The ANOVA detected a significant main effect of auditory condition, *F*(1, 111) = 13.33, *p* = 0.00038, *η*^2^ = 0.35. Post hoc Tukey tests revealed that the score for this subscale was significantly higher with sound than without. The ANOVA also detected a significant two-way interaction of auditory condition and VSS condition, *F*(1, 111) = 3.40, *p* = 0.048, *η*^2^ = 0.09. Post hoc Tukey tests revealed that the E+S+ group scored the Evaluative subscale significantly higher than did the other three groups. There was no significant main effect of VSS condition or gender, nor any other significant two- or three-way interactions for the Evaluative subscale.

**Table 1:** ANOVA predicting the Evaluative subscale of the Sexual Arousal and Desire Inventory

Term	*df*	MS	*F*	*p*
Auditory (S+ vs. S−)	1, 111	5486.81	13.33	**0.00038**
Version (E+ vs. E−)	1, 111	144.41	0.35	0.555
Gender	1, 111	40.82	0.10	0.753
Auditory*Version	1, 111	1644.94	3.98	**0.048**
Auditory*Gender	1, 111	154.14	0.37	0.542
Version*Gender	1, 111	109.88	0.27	0.606
Auditory*Version*Gender	1, 111	101.98	0.25	0.620

*p* values in bold represent statistically significant effects

### Physiological Subscale

Table 2 shows the results of the ANOVA for the Physiological subscale ratings of the four videos by women and men. The ANOVA detected a significant main effect of auditory condition, *F*(1, 111) = 13.40, *p* = 0.00039, *η*^2^ = 0.35. Post hoc Tukey tests revealed that the score for this subscale was significantly higher with sound than without. None of the other main effects or interactions were significant.

**Table 2:** ANOVA predicting the Physiological subscale of the Sexual Arousal and Desire Inventory

Term	*df*	MS	*F*	*p*
Auditory (S+ vs. S−)	1, 111	2660.53	13.40	**0.00039**
Version (E+ vs. E−)	1, 111	3.57	0.02	0.894
Gender	1, 111	18.67	0.09	0.760
Auditory*Version	1, 111	6.05	0.03	0.862
Auditory*Gender	1, 111	133.20	0.67	0.415
Version*Gender	1, 111	85.35	0.43	0.513
Auditory*Version*Gender	1, 111	234.76	1.18	0.279

*p* value in bold represent statistically significant effects

### Motivational Subscale

Table 3 shows the results of the ANOVA for the Motivational subscale ratings of the four videos by women and men. The ANOVA detected a significant main effect of auditory condition, *F*(1, 111) = 5.30, *p* = 0.023, *η*^2^ = 0.14. Post hoc Tukey tests revealed that the score for this subscale was higher with sound than without. The ANOVA also detected a trend toward a two-way interaction of auditory condition and VSS condition, *F*(1, 111) = 3.26, *p* = 0.073, *η*^2^ = 0.08. Post hoc Tukey tests revealed that the E + S + group scored the Motivational subscale higher than did the other three groups. None of the other main effects or interactions were significant.

**Table 3:** ANOVA predicting the Motivational subscale of the Sexual Arousal and Desire Inventory

Term	*df*	MS	*F*	*p*
Auditory (S+ vs. S−)	1, 111	406.78	5.303	**0.023**
Version (E+ vs. E−)	1, 111	0.01	< 0.01	0.996
Gender	1, 111	36.66	0.48	0.491
Auditory*Version	1, 111	250.26	3.26	***0.074***
Auditory*Gender	1, 111	7.45	0.10	0.756
Version*Gender	1, 111	102.99	1.34	0.249
Auditory*Version*Gender	1, 111	69.85	0.91	0.342

*p* value in bold represent statistically significant effects. *p* value in bold italic represent trends toward significance (between 0.05 and 0.1) with a moderate to high effect size

### Negative/Aversive Subscale

Table 4 shows the results of the ANOVA for the Negative/Aversive subscale ratings of the four videos by women and men. The ANOVA detected a significant three-way interaction of auditory condition, VSS condition, and gender, *F*(1, 111) = 5.49, *p* = 0.02, *η*^2^ = 0.14. Post hoc Tukey tests revealed that women and men did not differ in their ratings of the auditory condition with sound across the VSS conditions. However, in the auditory condition without sound, women found the E+ condition significantly more Negative/Aversive than men, but found the E− condition significantly less Negative/Aversive than men. The ANOVA detected a trend toward a significant two-way interaction of VSS condition and gender, *F*(1, 111) = 3.12, *p* = 0.08, *η*^2^ = 0.08. Post hoc Tukey tests revealed that men rated the E- condition without sound lower than with sound, whereas women rated the E + condition higher without sound than with sound. There were no significant main effects or other significant interactions.

**Table 4:** ANOVA predicting the Negative/Aversive subscale of Sexual Arousal and Desire Inventory

Term	*df*	MS	*F*	*p*
Auditory (S+ vs. S−)	1, 111	321.24	1.96	0.165
Version (E+ vs. E−)	1, 111	91.77	0.56	0.456
Gender	1, 111	18.79	0.11	0.736
Auditory*Version	1, 111	166.56	1.01	0.316
Auditory*Gender	1, 111	450.31	2.74	0.101
Version*Gender	1, 111	512.01	3.12	***0.080***
Auditory*Version*Gender	1, 111	902.83	5.49	**0.021**

*p* value in bold represent statistically significant effects. *p* value in bold italic represent trends toward significance (between 0.05 and 0.1) with a moderate to high effect size

## Discussion

The present study examined whether subjective assessments of sexual arousal and desire provoked by a sexually explicit video would be altered if the video was presented with or without the soundtrack, and with or without the explicit ejaculation scene that terminated the sexual interaction. As hypothesized, the video without sound was rated significantly lower overall on the Evaluative, Physiological, and Motivational subscales of the SADI. However, we also hypothesized that the video without sound might be rated higher overall on the Negative/Aversive subscale, which did not happen. This indicates that the positive and negative aspects of the video were assessed independently of one another, and that overall, the addition of the soundtrack which contained music and emotional/sensual utterances mostly of the female actor, increased the subjective arousal experienced during the video.

A gender difference was also found in the present study when comparing the ejaculatory ending with and without sound. Without sound, the ejaculatory ending was rated significantly higher on the Negative/Aversive subscale by women, compared to men, whereas the lack of the ejaculatory ending was rated higher on this subscale by men relative to women. In contrast, with sound, there were no significant differences between women and men for the videos with or without the ejaculatory ending. Interestingly, the ejaculation by the male actor was responded to by the female actor with a smile, immersion of her fingers in the ejaculate, and emotional utterances of satisfaction. The lack of emotional utterances likely determined whether women found the video without sound to be viewed more negatively than with sound. This suggests that her auditory cues signaled a clear and gratifying end to the sexual interaction, which lessened the negative impact of the ejaculation scene. However, for men, the sexual interaction without an ejaculatory ending was rated more negatively without sound than with sound, suggesting that the lack of auditory cues that had already diminished the positive rating of arousal to the video also made the version without ejaculation more negative for the men. It is interesting to note that the version with sound and ejaculation was rated somewhat higher by women on the Evaluative subscale than the version with sound but without ejaculation. This suggests that cultural changes consonant with greater exposure to sexually explicit videos on the internet may be reducing the negative appraisal of male-centric content for heterosexual women. In addition, we note that all of the incomplete SADI questionnaires were returned from women and men in the two groups without sound. Although there is no good explanation for this, it is possible that the participants who did not complete the SADI found the video without sound considerably less arousing or engaging. In sum, these data suggest that that ASCs contribute importantly to the interpretation of context, degree of absorption, and sexual satisfaction for both women and men.

The video used in the present study had been rated previously by both women and men as inducing a moderate level of subjective sexual arousal (Janssen et al., 2003). This was also found in the present study. Ratings of the video on the Evaluative, Physiological, and Motivational subscales of the SADI were between 30 and 50% of the total possible rating scores for each subscale, depending on the version of the video. This is remarkable in that the video was made in the 1990s, and the original version was responded to similarly by a different, more internet-savvy and porn-experienced generation of university-aged participants. It is unclear to what extent verbal and/or non-verbal ASCs might contribute to subjective sexual arousal provoked by a video of higher or lower incentive value. However, a previous study utilized a male-centric fantasy gang rape video sequence in which the female actor initially “rejects” sexual activity but then appears to enjoy it as the scene progresses (Pfaus et al., 1986). The clip with the original soundtrack (rock music, dialog, and both verbal and non-verbal emotional utterances) was rated by university-aged males as significantly more “pornographic” (more negative connotation) and less “erotic” (more positive connotation) than the same clip with the soundtrack replaced with relaxing background music, which was rated less “pornographic.” In addition, ratings of subjective sexual arousal correlated significantly with ratings of erotic, but not pornographic, content. Although it could not be determined in that study which aspects of the original soundtrack may have led to a more negative assessment, it is likely that the dialog and initial emotional verbal and non-verbal utterances of rejection and fear by the female actor created a mismatch of arousal, tension, and aversion in the viewer. As mentioned above, it is likely that the positive emotional non-verbal utterances of the female actor in the video used in the present study led to higher positive and less negative ratings for both women and men.

Although women are generally better than men at assessing emotional prosody from audio recordings alone (Fujisawa & Shinohara, 2011; Lambrecht et al., 2014; Schirmer et al., 2005; Waaramaa, 2017), the relative ability to accurately detect emotion from non-verbal utterances depends on the particular emotion (Lin et al., 2021). In the Lin et al. study, women were faster and more accurate than men at detecting happiness and sadness from non-verbal utterances, whereas men were faster and more accurate than women at detecting anger from the same sources. The present findings underscore the possibility that women and men are affected similarly by the non-verbal auditory qualities of a sexually explicit video; however, for women, the presence of ASCs changed how they were affected by other aspects of the film (i.e., during the depiction of explicit ejaculation). There was no verbal dialog in the video, only repeated non-verbal emotional utterances by the female actor and a grunt at the end by the male actor. It is possible that experience with sexually explicit VSS and ASCs establishes an expectation of actions and sounds, especially verbal and non-verbal emotional utterances of the actors that allow viewers to infer positive or negative emotional valence. Indeed, experience with emotional vocalizations through the lifespan makes older individuals better able to infer correct emotional reactions compared to younger individuals (Fecteau et al., 2005).

The present study examined the role of ASCs and a male-centric ejaculation scene in only one video that induced moderate arousal, which limits the interpretation of the effects. It would be interesting in the future studies to examine the category specificity of female and male reactions to other types of ASCs, especially if the accompanying VSS is more or less arousing, or portrays different sexual interactions that are consistent or inconsistent with people’s orientations or preferred types of expression. It is possible that positively hedonic ASCs could have a compensatory effect on VSS that depict violent or non-consensual interactions, or vice versa, if the ASCs depict aversive prosody during an otherwise positive visual depiction of sexual interaction. Future studies should also consider the potentially different salience of ASCs alone in sighted and visually-impaired individuals.

### Conclusion

Sexually explicit visual materials exist to induce and/or enhance fantasy and sexual arousal during solo or partnered sexual activity. The present study shows that the addition of auditory cues from an erotic video soundtrack enhances the subjective perception of sexual arousal and desire equivalently in women and men in response to VSS. Conversely, the lack of sound, especially pleasurable vocalizations, makes it difficult for participants to assess the valence of particular sexual interactions, especially those that could be assessed negatively, such as an explicit male-centric scene of ejaculation on the breasts of a female actor. It is not clear whether the lack of a soundtrack with emotional vocalizations in some previous studies may have contributed to a lessening of subjective and physiological sexual arousal, or to what extent a soundtrack with only music and/or poorly-executed dialog and fake vocalizations may have had (or continues to have) the same effect. This has serious implications for understanding psychological processes and neural systems activated by sexually explicit materials, and is an important consideration when attempting to generate a common set of optimal, validated stimuli that can be used in research on sexual arousal and desire.
